# The environment of professional practice and Burnout in nurses in primary healthcare

**DOI:** 10.1590/0104-1169.0011.2497

**Published:** 2014

**Authors:** Vera Regina Lorenz, Edinêis de Brito Guirardello

**Affiliations:** 1 PhD, Professor, Colégio Técnico de Campinas, Universidade Estadual de Campinas, Campinas, SP, Brazil; 2 PhD, Professor Associado, Faculdade de Enfermagem, Universidade Estadual de Campinas, Campinas, SP, Brazil

**Keywords:** Health Facility Environment, Burnout, Professional, Working Conditions, Job Satisfaction, Primary Health Care, Nurses

## Abstract

**OBJECTIVES:**

to assess how nurses perceive autonomy, control over the environment, the professional relationship between nurses and physicians and the organizational support and correlate them with burnout, satisfaction at work, quality of work and the intention to quit work in primary healthcare.

**METHOD:**

cross-sectional and correlation study, using a sample of 198 nurses. The tools used were the Nursing Work Index Revised, Maslach Burnout Inventory and a form to characterize the nurses. To analyze the data, descriptive statistics were applied and Spearman's correlation coefficient was used.

**RESULTS:**

the nurses assessed that the environment is partially favorable for: autonomy, professional relationship and organizational support and that the control over this environment is limited. Significant correlations were evidenced between the Nursing Work Index Revised, Maslach Burnout Inventory and the variables: satisfaction at work, quality of care and the intent to quit the job.

**CONCLUSION:**

the nurses' perceptions regarding the environment of practice are correlated with burnout, satisfaction at work, quality of care and the intent to quit the job. This study provides support for the restructuring of work processes in the primary health care environment and for communication among the health service management, human resources and occupational health areas.

## Introduction

Basic or primary health care is considered the primary level of contact between individuals, families and communities and the Unified Health System (SUS) and family health is the strategy to reorganize is, considered as an alternative health care model to achieve universalization, equity and integrality^(^ 1 ^)^.

On the other hand, as a set of individual and collective health actions^(^ 1 ^)^, primary health care is an important nursing activity area, where the nurses develop and articulate actions to promote, prevent and recover the population's health^(^ 2 ^)^.

In the practice sphere, the nurses work independently and interdependently in teams^(^ 1 ^)^, with actions centered on the organization and management of health work processes for individual care. At the same time, there is a need to organize work processes focused on care for family needs and the qualification of care for individuals in their family and community contexts^(^ 3 ^)^. That imposes challenges for the nurses in daily practices, as different work processes demand more cognitive efforts and increase the work loads^(^ 4 ^)^, making the workers' actual activities more complex.

Another important aspect that puts a strain on the nurses in primary health care is their role as team leaders, involving the coordination of community health agents and the nursing team's work, besides specific and shared care activities, such as: nursing consultations and procedures, educational activities, home visits and surveillance actions^(^ 5 ^)^.

Nursing practice is characterized by the dichotomy between care and management actions and by tensions deriving from the task division in collective work management, resulting in conflicts with the physicians and with central management, in which the nurses perceive the unequal accountability as a work burden^(^ 6 ^)^.

Nurses working in environments that are considered favorable to professional practice, with sufficient human and material resources, describe positive experiences at work and a better perceived quality of care^(^ 6 ^)^. In addition, there is evidence that autonomy, cordial professional relationships between nurses and physicians, control over the professional practice environment and organizational support are environmental attributes that, when perceived by the nurses, enhance professional practice, guarantee greater satisfaction and contribute to a better quality of care offered to patients in inpatient services^(^ 7 ^-^ 9 ^)^. Nevertheless, there are no studies in Brazil that assess these favorable characteristics in the professional practice environment of nurses in primary healthcare, which justifies the development of this study.

In health, the restructuring of the care production produces different care models that imply different organizational dynamics^(^ 10 ^)^, which influence the work processes of the nurses who play an important role in the reorganization of the care model within the health surveillance logic, which requires new technical and interpersonal skills to exercise their actual work activities, enhancing the complexity of these professionals' attributions and responsibilities.

On the other hand, the work environment in primary health care is not always favorable to the professional practice of nurses and, often, the physical environment is inappropriate; human resources, equipment and inputs are insufficient^(^ 11 ^)^; the professionals are exposed to different categories of risks to health and occupational safety problems in the work environment^(^ 12 ^)^ and to different forms of violence^(^ 12 ^-^ 13 ^)^; the staff turnover levels are considerable^(^ 14 ^)^; the remuneration is low; the professionals choose to have more than one job, compromising the quality of work and their own health.

In work situations in which the nurses and continuously exposed to mental burdens, the environmental factors that are perceived with displeasure and without effective coping mechanisms, burnout is evidenced^(^ 15 ^)^. This is a three-dimensional syndrome, characterized by emotional exhaustion, depersonalization and reduced professional accomplishment, whose dimensions are independent and mutually related^(^ 16 ^)^.

Burnout can be considered a work-related disease because it is associated with exposure to health and occupational safety risk factors present in the work environment. In that sense, in a Brazilian study of nurses working at a public hospital, it was evidenced that the increase of perceived stress is significantly correlated with burnout^(^ 15 ^)^, while it was identified in a Canadian study that emotional burnout is positively associated with the work demands^(^ 17 ^)^.

While health, wellbeing and quality of life at work have been identified as key issues in the recruitment and retention of nurses, absenteeism and the phenomenon of the lack of human resources in nursing have made the professionals eliminate days of leave, making them more prone to physical and mental health problems due to the workload they carry^(^ 18 ^)^, besides preventing them from offering high-quality care the whole time, weakening their performance and distancing them from specific work environments or from their profession^(^ 17 ^)^.

To allow the nurses to contribute to the reorganization of primary healthcare according to the logical of the health surveillance model, without a continuing feeling of burnout, treating patients and co-works in a humanized manner the whole time, feeling accomplished and satisfied at work, perceiving the quality of the work as good and moving away from the intent to quit their job, it is important for them to perceive that they have: autonomy, control over the environment of practice, a cordial professional relationship between nurses and physicians and organizational support, for them to assess the environment as favorable to innovative professional practices, which the ideal healthcare model requires to be effective.

In that context, the objective in this study is to analyze how nurses in primary healthcare perceive the autonomy, control over the environment of practice, cordial professional relationship between nurses and physicians and organizational support and correlate these perceptions with the dimensions of the burnout syndrome, professional satisfaction, perceived quality of care and intention to quit the current job.

## Method

A cross-sectional correlation study was undertaken in the primary healthcare services of the public network in a Brazilian city that is considered fully in charge of its municipal health system, with an estimated population of 1,144,862 inhabitants according to the Brazilian Institute of Geography and Statistics for 2013. The study population consisted of 287 nurses, obtained from a list of the Municipal Health Secretariat in February 2012, who complied with the criteria for inclusion in the sample: working as a nurse at one of the primary healthcare services at the time of the data collection, i.e. between June and December 2012. Nurses who were on holiday, leave or assigned to other services during the data collection period or who were unwilling to participate in the research were excluded. Thus, the sample resulted in 198 nurses.

Approval for the research project was obtained from the research ethics committee under number 573/2011, with the agreement of the municipal health secretariat. The subjects who accepted to participate in the study received an individual enveloped with a characterization form, the research tools to be answered, a pen and a seal to guarantee the participants' anonymity and the free and informed consent form, which the researcher and participant signed in two copies.

To collect the data, the following tools were used: the Brazilian version of the Nursing Work Index Revised (NWI-R)^(^ 8 ^-^ 9 ^)^; the Brazilian version of the Maslach Burnout Inventory (MBI), adapted and validated by Tamayo^(^ 19 ^)^, which has been used to assess burnout in inpatient services^(^ 8 ^-^ 9 ^)^, and a personal and professional characterization form, to which three questions were added to assess: professional satisfaction, perceived quality of care and intention to quit the current job^(^ 8 ^-^ 9 ^)^.

The Brazilian version of the NWI-R contains 57 items and its objective is to measure the nurses' perception of favorable characteristics in the professional practice environment, as follows: autonomy, control over the professional practice environment, cordial professional relationship between nurses and physicians and organizational support^(^ 8 ^-^ 9 ^,^ 20 ^)^. For analysis purposes, only 15 items have been considered sufficient^(^ 8 ^-^ 9 ^,^ 20 ^)^, which are distributed among four subscales: autonomy (five items), cordial professional relationship between nurses and physicians (three items), control over the professional practice environment (seven items) and organizational support (ten items), which contain the same items as the previous subscales^(^ 8 ^-^ 9 ^,^ 20 ^)^. The NWI-R measure is a Likert scale ranging from one (I completely agree) to four (I completely disagree) and, the lower the score, the greater the perceived: autonomy, control over the professional practice environment, cordial professional relationship between nurses and physicians and organizational support, indicating an environment that is favorable to practice^(^ 8 ^-^ 9 ^,^ 20 ^)^.

The MBI assesses how the workers experience their work through the frequency of feelings related to the burnout syndrome^(^ 16 ^-^ 19 ^)^. The inventory contains 22 items, distributed in three subscales: emotional exhaustion (nine items), depersonalization (five items) and personal accomplishment (eight items)^(^ 18 ^,^ 21 ^)^. The MBI measures is a Likert scale ranging from one (never) to five (always)^(^ 19 ^)^ and, the higher the score, the more frequent are feelings of emotional exhaustion, depersonalization and personal accomplishment^(^ 16 ^-^ 19 ^)^. High scores on the emotional exhaustion and depersonalization subscales and low scores on the personal accomplishment subscale indicate a high level of burnout^(^ 16 ^-^ 19 ^)^.

Satisfaction at work was measured by means of a Likert scale ranging from one (highly satisfied) to four (highly dissatisfied)^(^ 8 ^-^ 9 ^)^. The perceived quality of care was measured on a Likert scale from one (very bad) to four (very good)^(^ 8 ^-^ 9 ^)^. The intent to quit the current job was measured using a visual analogue scale from zero (none) to 100 (a lot) millimeters.

For the data analysis, the software Statistical Analysis System (SAS) version 9.2 was used. For the descriptive analysis, means, standard deviations (SD), medians, minimum, maximum and percentages were used. The reliability was assessed using Cronbach's alpha coefficient and values superior to 0.60 were considered satisfactory^(^ 21 ^)^.

To analyze the existence of correlations, Spearman's correlation coefficient was used and significance was set at 5% (p<0.05). To study the correlations between the NWI-R subscales and the MBI subscales, the answers of 168 nurses were considered who answered all items of both tools' subscales. To analyze the existence of correlations between the NWI-R subscales and the variables: satisfaction at work, perceived quality of care and the intent to quit the current job, the answers of 168, 165 and 162 nurses were calculated, who answered the respective variables and all items of the NWI-R subscales.

## Results

The sample consisted of 88.4% of female nurses; 46.2 % single, 43.1% married, 9.2% separated and 1.5% widowed. The mean age was 36.3 years (SD±10.4; min=23 and Max=61 years). The time since graduation was equal to 10.6 years (SD±9.1, min=2 and max=35 years); length of work experience in municipal government 6.6 years (SD±7.7, min=0.2 and max=28.2 years) and 4.9 years of work at the current service (SD±6.3, min=0.1 and max=25.5 years).

Most of the nurses (59.4%) declared that they held a *lato sensu *graduate degree in collective health; 13.7% in the hospital area; 6.1% in other areas. Four nurses (2.0%) indicated they held a Master's degree in nursing, one (0.5%) in collective health and three (1.5%) were taking a Master's course.

As regards the employment contract, the majority (98.5%) declared working under the statutory regime and 1.5% under the regime of the consolidation of Labor Laws (CLT) under a contract of undetermined length. Among both, 9.7% declared they had more than one employment contract. The mean weekly workload, including the other job, was 38.3h (SD±8.0, min=30.0 and max=88.0). Concerning the work shift, 43.2% worked mixed shifts (morning and afternoon or morning and night or afternoon and night), 29.7% afternoon, 25.5% morning and 1.5% night.

As regards the satisfaction at work, the majority (62.6%) considered they were satisfied, 34.9% dissatisfied, 1.5% highly dissatisfied and 1.0% highly satisfied. With regard to the perceived quality of care delivered to users at the service, 79.0% considered it was good, 13.3% bad, 7.2% very good and 0.5% very bad. For the variable intention to quit the current job, the mean result was 28.4 millimeters (SD± 27.0; min=00.0 and max=100.0).

The mean perception about the nurses' professional practice environment bordered on two points for the subscales: autonomy, professional relationship between nurses and physicians and organizational support; and bordered on three points for the subscale control over the professional practice environment. As regards the internal consistency, a Cronbach's alpha coefficient superior to 0.60 was found for the subscales: control over the practice environment, cordial professional relationship between nurses and physicians and organizational support and 0.52 for the subscale autonomy (Table 1).

**Table 1:** Mean, standard deviation, mediation, variation and Cronbach's alpha coefficient of the subscales of the Nursing Work Index Revised (n=168). Campinas, SP, Brazil, 2012

Subscales of the Nursing Work Index Revised	Mean	Standard Deviation	Median	Variation Min.-Max.	Cronbach’s alpha
Autonomy	2.1	0.5	2.0	1.0-3.2	0.52
Control over practice environment	2.7	0.5	2.6	1.4-3.9	0.66
Nurse-physician relations	1.9	0.6	2.0	1.0-3.7	0.78
Organizational support	2.2	0.4	2.2	1.1-3.3	0.70

The mean frequency of the feelings related to the burnout syndrome was 24.6 for emotional exhaustion, 9.4 for depersonalization and 30.4 for reduced personal accomplishment and the reliability ranged between 0.66 and 0.86 (Table 2).

**Table 2:** Mean, standard deviation, median and Cronbach's alpha coefficient of the subscales of the Maslach Burnout Inventory (n=168). Campinas, SP, Brazil, 2012

Subscales of the Maslach Burnout Inventory	Mean	Standard Deviation	Median	Variation Min.-Max.	Cronbach’s alpha
Emotional exhaustion	24.6	5.7	25.0	11.0-43.0	0.86
Depersonalization	9.4	3.0	9.0	5.0-20.0	0.66
Reduced personal accomplishment	30.4	3.5	30.5	20.0-38.0	0.72

In the evaluation of the existence of correlations between the subscales of the NWI-R and the MBI, the data showed that the subscales autonomy, control over the practice environment and organizational support of the NWI-R showed eight significant correlations with the MBI subscales emotional exhaustion, depersonalization and personal accomplishment (Table 3).

**Table 3:** Spearman correlation coefficient between subscales of the Nursing Work Index Revised and the Maslach Burnout Inventory subscales (n=168). Campinas, SP, Brasil, 2012

Subscales of the Nursing Work Index Revised	Subscales of the Maslach Burnout Inventory		
	Emotional exhaustion	Depersonalization	Personal accomplishment
Autonomy	0.17*	0.14	-0.25^†^
Control over practice environment	0.41^†^	0.18*	-0.18*
Relation between nurse and physician	0.04	0.13	-0.08
Organizational support	0.32^†^	0.21†	-0.24^†^

* p<0.05

† p<0.01

In addition, the existence of correlations was assessed between the NWI-R subscales and the variables: satisfaction at work, perceived quality of care and intention to quite the current job, showing seven significant correlations (Table 4).

**Table 4:** Spearman correlation coefficient between subscales of the Nursing Work Index Revised and the variables satisfaction at work, perceived quality of care and intention to quite the current job. Campinas, SP, Brazil, 2012

Subscales of the Nursing Work Index Revised	Variables		
	Satisfaction at work (n=168)	Quality of care (n=165)	Intention to quit job (n=162)
Autonomy	-0.11	-0.20*	0.22^†^
Control over practice environment	-0.30^†^	-0.18*	0.15
Relation between nurse and physician	-0.03	-0.18*	-0.01
Organizational support	-0.27^†^	-0.26^†^	0.13

* p<0.05

† p<0.01

## Discussion

The nurses in this study are predominantly female, which characterizes the nursing workforce, justified by the socio-historical trajectory of the profession. They are young adults, with a mean 36 years of age, mostly single or married.

The majority holds a graduate degree in collective health, which demonstrates commitment to the quality of their professional performance, favorable to the achievement of good results. It is highlighted that the work experience of more than six years in primary care and the length of experience of more than four years at the current service indicates a group of experienced and professionally mature nurses.

The fact that the majority is working under the statutory regime can be attributed by the CLT nurses' decision not to participate in this research, whose job contracts of limited length were about to expire or had expired, causing a possible selection bias.

The weekly hour load in this city is 36 hours and the corresponding mean was two hours longer, which can derive from the need for more nursing hours at the service and the professionals' option to work overtime, generating additional salary. Overtime means fewer hours for rest, family life and other activities needed to achieve quality of life.

The majority declares they are satisfied with their work and considers the quality of care offered to the users good. On the other hand, the intention to leave their current job was identified among the nurses, which can culminate in the intention to leave the profession in function of the work conditions^(^ 14 ^-^ 17 ^)^.

The nurses assessed the work environment as partially favorable to practice, ranging from 1.9 to 2.2 for the NWI-R subscales: cordial professional relationship between nurses and physicians, autonomy and organizational support, on a scale ranging from one (favorable environment) to four (unfavorable environment) (Table 1). As regards the perceived control over the practice environment, the mean score of 2.7 points demonstrates that the nurses have little control over the environment they work in (Table 1). 

Little autonomy, little control over the environment and little organizational support may reflect two existing care models, in which the nurses develop activities related to traditional primary care, focused on the user's disease and guided by medical-curative interventions (traditional technology); at the same time as their activities related to the expansion and consolidation of the family health strategy^(^ 10 ^)^, focused on the family and its social relations, guided by the SUS principles (technological innovation). These imply different work processes, demand more cognitive efforts and increase the workloads the nurses have to carry, causing dissatisfaction and stress^(^ 4 ^)^.

The increased workloads for the nurses who continue working, associated with the reduced perceived control over the environment, autonomy and organizational support, represent one of the consequences of the underfinancing of the SUS at the state and federal levels. Associated with the fiscal responsibility law, this compels the cities to limit their spending on health staff, resulting in precarious work relations, health work management and work conditions^(^ 11 ^)^. For primary health care, this situation takes the form of the non holding of public competition procedures for statutory functions and insufficient nursing staff dimensioning for the population's needs, putting a burden on the insufficient number of professionals who continue working. As a measure to reduce the insufficient number of staff in the short term, statutory nursing functions are replaced by non-statutory functions under the CLT regime, whose limited-length contracts can be extended for two years at most, compromising the bonding with the users, teamwork and intersectoral work.

In the practice context, where the nurses work independently and interdependently in teams^(^ 1 ^)^, the work processes require additional efforts, such as strategy development, preview of future events and intellectual efforts to solve problems and overcome difficulties^(^ 22 ^)^. Efforts are highlighted to overcome the psychological repercussions and mental suffering the exposure to different forms of work violence, violence at work and indirect violence^(^ 12 ^-^ 13 ^)^.

As regards the reliability of the NWI-R, the Cronbach's alpha coefficients were considered satisfactory for the cordial professional relationship between nurses and physicians, control over the professional practice environment and organizational support.

Based on the evidences of eight significant direct and inverse correlations between the NWI-R and MBI subscales, it can be inferred that, the lesser the perceived autonomy, the more frequent the emotional exhaustion and the less frequent the personal accomplishment. The lesser the control over the practice environment and the organizational support, the more frequent are the feelings of emotional exhaustion and depersonalization and the less frequent the feeling of personal accomplishment will be. Only the subscale cordial professional relationship between nurse and physician was not significantly correlated with the MBI subscales, which can be justified by the specificity of the subscale, which does not represent all of the required interpersonal relations for multiprofessional and intersectoral teamwork in primary healthcare^(^ 1 ^)^.

Another important aspect of these results refers to the existence of seven significant correlations between the NWI-R subscales and the variables: satisfaction at work, perceived quality of care and intention to leave the current job. The worse the perceived professional relation between nurse and physician, the worse the perceived quality of care. The worse the perceived control over the environment of professional practice and organizational support, the worse the perceived quality of care and the worse the satisfaction at work.

These findings are relevant for nurses, coordinators and health managers to reassess the different aspects involving the nurses' practice environment in primary healthcare, with a view to creating conditions that favor safe practice environments, with positive repercussions for professionals and a better quality of care for SUS users. Hence, it is of interest to society to offer environments favorable to the nurses' professional practice in primary healthcare in order to recruit and retain more nurses - essential professionals who contribute to the accessibility and use of the health services, to the longitudinality and integrality of care and the articulation among the different services^(^ 1 ^,^ 23 ^)^, generating the professionals and SUS users' satisfaction. This study offers support for the restructuring of work processes and practices in the primary care context and for the communication among the health service management and occupational health areas.

## Conclusions

The study permitted assessing the nurses' professional practice environment in primary healthcare and the nurses assessed it as partially favorable to practice, represented by most of the NWI-R subscales: autonomy, cordial professional relations between nurse and physician and organizational support, except for the subscale that represents the control over the environment of practice which, due to the nurses' limited perception, indicated that the environment is partially unfavorable to practice for this variable.

The nurses' perceptions about the attributes of the practice environment, according to the NWI-R variables, are correlated with burnout and three variables: satisfaction at work, quality of care and intention to quit the current job.

As regards the correlations between the perceived environment and burnout, it is concluded that, when the perceived autonomy is limited, the frequency of feelings of emotional exhaustion increases and the frequency of feelings of personal accomplishment drops; when the perceived control over the environment of professional practice is reduced, the frequency of feelings of emotional exhaustion and depersonalization increases and the frequency of feelings of personal accomplishment drops.

Concerning the correlations between the perceptions regarding the environment and the three variables, it is concluded that: reducing the perceived autonomy reduces the perceived quality of care and enhances the intent to quit the current job; reducing the perceived control over the environment of professional practice reduces the satisfaction at work and perceived quality of care; reducing the perceived cordial professional relationship between nurse and physician decreases the perceived quality of care; reducing the perceived organizational support reduces the satisfaction at work and perceived quality of care.

This study contributes to support reflections on the nurses' environment of practice in primary healthcare and to restructure the work processes according to the health surveillance model, so as to turn work into a source of pleasure and accomplishment.

## Study limitations

Among the study limitations, the sample size is highlighted, which represented 69.0% of the population of primary healthcare nurses, the possible selection bias and the impossibility to generalize the results, as the study was restricted to one city in the state of São Paulo. Therefore, further research is needed, involving nurses from different regions of the state and of Brazil.
